# Difficult Patient Behaviors in the Hospital Setting: A Narrative Review

**DOI:** 10.1007/s11606-025-10078-8

**Published:** 2025-12-10

**Authors:** Alyssa C. Smith, Danielle K. Patterson, Emily G. Holmes

**Affiliations:** 1 Department of Psychiatry, Indiana University School of Medicine, Indianapolis, IN USA; 2 Fairbanks Center for Medical Ethics at Indiana University Health, Indianapolis, IN USA

**Keywords:** difficult behaviors, acute medical, hospital

## Abstract

In the inpatient medical setting, difficult patient behaviors can complicate care delivery. When difficult behaviors are present, patients may experience worsened outcomes, and medical providers may experience distress. Most recommendations for such behaviors in the literature focus on outpatient care or inpatient psychiatric settings; recommendations for inpatient medical settings are limited. While difficult behaviors are commonly associated with personality disorders, patients without AQ6personality disorders may also exhibit these behaviors when acutely stressed, such as when they are experiencing a medical illness. Common difficult behaviors include splitting, acting out, avoidance, boundary crossing, false compliance, and dependence. Increased recognition of difficult behaviors, understanding of why these behaviors occur, and familiarity with potential management techniques may improve patient outcomes and reduce clinician distress. We provide a narrative review of these challenging behaviors, discuss potential underlying causes, and provide management recommendations.

## INTRODUCTION

Difficult patient behaviors can complicate and interrupt patient care, resulting in increased morbidity, mortality, and costs—both in terms of healthcare costs and in loss of productivity.^1–4^ Additionally, these behaviors tend to elicit countertransference reactions from medical teams and contribute to clinician distress and burnout,^5^ further complicating care. Most studies on the prevalence of difficult behaviors have focused on the outpatient setting, with an estimated 15% of encounters being complicated by such behaviors.^6,7^ While inpatient studies are limited, a recent study of hospitalized patients found that approximately 25% were perceived as having difficult behaviors by their physician.^8^

Despite the significance and prevalence of these behaviors, many hospitals lack standardized approaches to mitigating disruptive behaviors,^9^ and much of the literature focuses on management in outpatient—not inpatient—settings.^5,10^ To help physicians familiarize themselves with these behaviors, the authors present a narrative review of these challenging behaviors, starting with a description of each, followed by potential patient motivations, common clinician responses, and management strategies (Table 1). As this is a narrative review and not scoping, not all behaviors or strategies will be discussed; instead, the most common will be highlighted and discussed. These include splitting, boundary crossing, acting out, malingering and sabotaging discharge, avoidance of care (also called “avoidant coping”), false compliance, and dependency.

**Table 1:** Challenging Behaviors that may be Encountered in the Hospital, Underlying Psychological Needs, and Suggested Management Techniques

Challenging behavior	Underlying psychological need(s)	Suggested management strategies
Splitting	Make sense of a complex situation Reduce anxiety Maintain self-esteem	Multidisciplinary team meeting(s) Joint rounds Team education Consistent approaches
Boundary crossing	Feel closer to physician Maintain control	Seek peer supervision Affirm support while setting limits
Acting out	Regain control	De-escalate situation Prioritize safety Set limits
Symptom falsification	Wanting to feel cared for Desire for attention and support	Consider confrontation Therapeutic discharge Avoid unnecessary workup and treatment
Avoidance	Need for encouragement and support Avoid emotional pain of a challenging situation	Assess for co-occurring syndromes (e.g., delirium) Provide adequate education Invite questions and participation
Dependence	Desire for connection Wanting to feel cared for	Provide choices Setting boundaries

## THE PHYSICIAN’S ROLE AND COUNTERTRANSFERENCE

Before discussing the individual difficult behaviors, it is important to acknowledge the social and cultural context of particular behaviors and the role of countertransference in these doctor-patient interactions. Importantly, behaviors and clinicians’ perceptions of behaviors are impacted and shaped by social and cultural contexts.^11^ For example, the behavior of Black patients may be perceived as “aggressive” more commonly than the behavior of White patients because of clinician bias.^12–14^ Cultural contexts must be considered before deeming a behavior inappropriate, and bias must be recognized and acknowledged. Additionally, understanding cultural contexts may help clinicians discuss and manage these behaviors with patients and families.

The role of the physician in the “difficult patient” interaction should not be overlooked. One study by Jackson and Kroenke demonstrated that the greatest predictor of experiencing a difficult patient encounter was the physician, not the patient.^6^ This is reassuring—if a physician can improve their response to challenging situations, they can reduce the burden of difficult interactions.

By reflecting on the behaviors that a physician finds particularly challenging and proactively considering the range of possible responses, the physician may feel better prepared to respond thoughtfully during a difficult patient interaction. To do so, one must understand and reflect on their countertransference. Countertransference, as classically defined by Sigmund Freud, is a therapist’s emotional response to a patient’s transference (or redirected feelings).^15,16^ Modernly, countertransference has been thought of as a healthcare provider’s total emotional response to a patient.^15,16^ For the purposes of this review, we will use the latter definition. Countertransference may include positive, neutral, or negative (such as frustration, anger, or distress) emotions and commonly involves mixtures of emotions. Importantly, countertransference can impact patient outcomes, particularly if the clinician has a strong positive or negative emotional response.^16^

## THE BEHAVIORS

### Idealization, Devaluation, and Splitting

Splitting is a defense mechanism in which a person divides experiences, objects, and people as either “all good” or “all bad,” with no in-between.^17^ Splitting is an attempt to understand complex situations, especially ones that are confusing or threatening. Patients may unconsciously use this defense to reduce anxiety and maintain self-esteem.

A common scenario on a medical floor occurs when a patient requests special privileges or benefits from team members, such as receiving medications early or via a particular route of administration, picking up snacks from the gift shop or food delivery services, or providing therapies at certain times of day or under certain circumstances. The team members who meet these requests become idealized, and those who do not are devalued, which in turn splits the team members against each other. Clinicians who meet the requests feel that they are providing the best care for the patient and turn against those who do not. The clinicians who do not meet the request become angered by the perceived permissiveness of those who do. The hospital is fertile ground for splitting dynamics due to frequent staff rotations and numerous aspects of care that can be negotiated.

To counteract this defense mechanism, one must first recognize that it is occurring and the countertransference evoked. For instance, the “good provider” may feel special and may be inclined to go “above and beyond” for the patient. The “bad provider” may feel rejected, hopeless, or mad; this may result in the clinician feeling inclined to avoid the patient, punish the patient, or even do less than medically indicated.^17,18^ Discussing such emotional responses should be done as a team and ideally include all clinicians and staff involved in the patient’s care.^18^

A multidisciplinary team meeting with physicians, nursing, social work, and other ancillary clinicians is crucial for bringing together the care team to identify and educate about the splitting that is occurring. The team can determine a consistent approach to addressing patient requests, then clearly present the plan to the patient to establish expectations; this can minimize further splitting.^19^ Joint rounds with all specialists involved with the patient’s care may also be helpful.^20^

### Boundary Crossing

Boundaries in physician-patient relationships separate professional behavior from unprofessional behavior and ethical behavior from unethical behavior. Therefore, any behavior that threatens the professional or ethical standards of the physician-patient relationship is a boundary concern. Issues are generally split into boundary crossings (a less serious infraction that may occasionally be therapeutic for the patient and does not cause harm) and boundary violations (a more serious breach of the relationship that is harmful for the patient).^21^ Patients may seek to breach professional boundaries in attempts to fulfill psychological needs, such as a need to feel closer to the physician or to exert control over a situation.

Most physicians appreciate how negative countertransference can adversely affect patient outcomes; however, positive countertransference can also be problematic. For example, a physician may make themselves available outside of their normal hours to a patient whom they like. Over time, the patient’s needs may grow, and the physician may become weary of responding to their increasing requests. The physician may become resentful of the patient or be faced with a difficult conversation to re-establish appropriate boundaries.

It may be therapeutic to explore these psychological needs with the patient. For example, when a patient asks their physician increasingly personal questions (for example, “how old are you?” and “are you married?”), a physician may counter by gently asking “what makes you ask me that?” Alternatively, a physician may respond to the questions by reaffirming the boundary (for example, “This visit is about you, not me. Let’s go back to talking about you and your symptoms so that I can best determine how to help you.”). In general, when physicians are considering disclosing personal details, they should ask themselves if the disclosure is expected to be therapeutic for the patient. If not, avoiding disclosure is recommended.

Similarly, a physician should carefully consider the potential outcomes before crossing the boundary of physical touch, such as a pat on the back or a hug. When patients are experiencing a large amount of distress, such as in acute grief or after receiving a serious diagnosis, boundary crossings may be beneficial.^22^ However, some patients may be inappropriately flirtatious; in these cases, we recommend against crossing physical boundaries. Seeking supervision from a colleague to obtain a second opinion and reaffirm boundaries may be helpful.^23^

In general, when a clinician finds themselves doing something for a patient they would not usually do, it is worthwhile to ask oneself the purpose of the action and consider whether the action is truly helpful or could be harmful. A physician may also consider whether a certain action is one that they would take for all patients—if not, this can be a warning signal that a specific action may be inappropriate.

### Acting Out

Acting out includes both physical aggression (towards staff or property) and verbal aggression (yelling, insulting, and threatening). Patients may exhibit hostile behavior in attempts to regain control of a situation. Anger, frustration, and fear are common countertransference responses to acting out behaviors; these emotions and subsequent behaviors resulting from these emotions tend to exacerbate the acting out behaviors.^24^ Key interventions for addressing acting out behaviors include establishing safety, recognizing and acknowledging the countertransference, and developing a plan to prevent the countertransference from further escalating the behavior.

When patients are physically aggressive, the physician should prioritize the safety of all parties, and hospital security should be involved. If available, medications for agitation should be administered. When the patient is verbally aggressive, providers may talk quietly and slowly to encourage the patient to lower their voice. If this is ineffective, it can be helpful to say something like, “I am unable to hear you when you are yelling.” If this is also ineffective, it is reasonable for the clinician to tell the patient they are leaving the room and will return when the situation has de-escalated. This is not the time to try to reason or argue with the patient. The physician should instead focus on de-escalating tactics. It can be helpful to see if the patient needs food, water, or medication for pain or other symptoms. The physician should pay close attention to their body language, ensuring an open posture with arms uncrossed and palms open.

After acting out has occurred, empathetically approaching and discussing behaviors with patients is a reasonable first step once the situation has fully deescalated.^25^ Exploring the underlying emotions and encouraging patients to find other outlets can prove beneficial.^24^ Limits may need to be set with the patient, with clear expectations for future behavior.^26^ When episodes of acting out occur repeatedly, care contracts or behavioral contracts may be considered. The Academy of Consultation-Liaison Psychiatry has a useful guide to creating such a care plan.^27^ Care contracts can vary widely across institutions. These contracts tend to be written agreements, with a copy provided to the patient, setting expectations for behavior, outlining rewards when behavioral expectations are met (e.g., an extra snack or TV time), and possibly including punishments when acting out occurs (e.g., restriction of phone privileges or TV time).

Care contracts have some notable limitations. These documents are not contracts as defined by law, and some may be perceived as punitive or infantilizing. Generally, the goal of a care contract should be to improve patient care, establish expectations of patients and clinicians, and address the underlying cause(s) and function(s) served by the problematic behavior.^27^ It may be helpful to focus on the behaviors of the medical team, rather than the patient’s actions. Ultimately, we are unable to control patients, and attempts to do so frequently escalate the situation and damage trust and rapport.

### Malingering and Factitious Disorder: Sabotaging Discharge

Both malingering and factitious disorder are characterized by intentional falsification or augmentation of symptoms for gain. In malingering, this gain is secondary and external, such as money, housing, or avoidance of legal charges. In factitious disorder, the gain is primary and subconscious, deriving from the psychological benefits of being in the sick role, such as feeling cared for or receiving attention and support.^28,29^ In many cases, this distinction can be difficult to discern, as patients may have both primary and secondary gains. An additional challenge is that patients usually do not admit to symptom falsification. Diagnosis of malingering and factitious disorder requires evidence of symptom falsification; physicians must take care to rule out medical causes of symptoms, especially in the absence of evidence of falsification.^29^ Common emotions provoked by suspected symptom falsification include frustration, anger, and dread.^30^

Unfortunately, due to the nature of these conditions, there is little evidence to guide physicians on how to address symptom falsification.^31^ The physician has several options for confronting the patient, ranging from more direct, forceful confrontation to a more persuasive confrontation that encourages the individual to seek psychiatric treatment. Another option is a more subtle attempt to allow the patient to “save face.”^32,33^ Many patients will request discharge once they have been confronted about falsified symptoms; therefore, confrontations must be carefully timed to ensure patient safety. Another tactic is directly discussing with the patient that no medical cause of the symptoms has been found, and as such, no further workup or treatment is indicated. In such cases, treatment is discharge from the acute setting with outpatient follow-up.

Providers may feel hesitant to discharge a patient who desires admission; Taylor et al.’s paper on the utility and necessity of the therapeutic discharge may be helpful in alleviating provider distress and provides a framework for this process.^34^ Recognition that unnecessary workup and admission come with the risk of financial and iatrogenic harm may be helpful to acknowledge. Even without a specific diagnosis, if further hospitalization is not indicated, discharge may be appropriate. When some patients are confronted with an upcoming discharge, they may “up the ante” to sabotage discharge and remain in the hospital. In these cases, 1:1 monitoring, camera monitoring, or involvement of security may be necessary to prevent self-inflicted harm.^34^ In most cases, patients with factitious disorder do not seek treatment for the factitious disorder itself. Therefore, the physician’s role may be limited to managing the sequelae of the falsified symptoms and reducing the potential for additional harm, either from the patient themselves or iatrogenic.

### Non-participation: Avoidance

Avoidance refers to when a patient does not engage in their care, which may present as refusing to engage with rehabilitative therapies, refusing needed testing, or minimally participating in conversations with the team. Avoidance may be driven by negative emotions. For instance, a patient may feel disrespected by their healthcare team or may feel insecure, either in being able to ask questions of their physicians or in the team’s competency.^35,36^ Additionally, patients may fear failure or may fear disappointing friends, family, or clinicians. Patients may lack the information needed to make a decision, particularly information that meets their level of healthcare literacy.^35–38^ Patients may decline treatment or fail to engage in treatment due to not fully understanding the severity of their condition or the potential benefits of the treatment. The physician may feel frustrated, angry, or resigned; these emotions can cause the physician to begin to become disengaged in the patient’s care themselves.^39^

Encouraging participation, such as by inviting the patient to ask questions or provide their thoughts and concerns, is a useful first step. Physicians can encourage and improve patient participation by using open-ended questions and patient-focused, collaborative, and supportive language.^37^ Ensuring that patients receive adequate education is necessary to improve participation if knowledge is a barrier, and handouts and consultation of services such as speech therapy may be useful. Tailoring the conversation to the specific patient (e.g., how it may impact their life, rather than how the condition affects people broadly) may be helpful, so that patients are adequately informed to decide based on their lives and values.^38^

The physician must also ensure that the patient does not have an underlying condition that impairs their ability to participate. For example, the patient may have a subtle hypoactive delirium limiting their ability to engage. Hypoactive delirium is under-recognized in the general hospital and can present with apathy, listlessness, decreased communication, and withdrawal.^40^ Catatonia is another neuropsychiatric diagnosis that may interfere with a patient’s ability to participate in medical care. If these conditions are suspected, a psychiatry consultation may be helpful for establishing a diagnosis and management plan.

### False Compliance

Non-adherence (also referred to as non-compliance) is the term used to describe a patient’s failure to follow medical recommendations. False compliance is a term used to describe the situation in which patients maintain that they are following their treatment plan despite evidence to the contrary. Examples of false compliance include a patient with heart failure who affirms that he is following his recommended diet, though his clinical course would suggest the contrary, and a patient with cystic fibrosis who does not appear to be following her pulmonary toilet routines based on repeated admissions and pulmonary function tests.

These behaviors may be guided by a variety of motivations, including feeling embarrassed or ashamed, not wanting to disappoint physicians, or to preserve autonomy.^41,42^ Patients may be non-adherent with medications and treatments because treatments can be seen as a symbol of illness or deficit, and medications can be seen as an imposition or stigmatizing. Additionally, some medications may have side effects that are intolerable or embarrassing to the patient (such as the sexual side effects of antidepressants), and openly exploring embarrassing side effects may be a fruitful avenue to allow the patient to acknowledge non-adherence and explore other options. There also may be some positive aspects of the illness, and patients may feel ambivalent about receiving treatment. Certain symptoms may be gratifying, provide compensation, relieve the patient of responsibilities, evoke caregiving from others, provide a sense of power, or serve other defensive functions.^43^

The physician may experience frustration because of the patient’s non-adherence or may doubt their own effectiveness. They may respond by becoming overly didactic or proscriptive. In situations of false compliance, the physician can feel stuck or ineffective, knowing that the patient is not adhering to treatment despite their assertions to the contrary. When non-adherence or false compliance is suspected, gentle confrontation may be helpful. Exaggerated questioning (e.g., asking, “How often do you miss your medication dosages? Every other day?” instead of asking, “Do you ever miss your medication dose?”) may facilitate an honest response by implying that less-than-perfect adherence is permissible. Similarly, giving the patient explicit permission to be imperfect (e.g., telling the patient, “It’s okay to miss dosages every now and then. To update your treatment plan, I need to know how often it’s occurring.”) may help the patient feel safe in their disclosure and encourage partnership in the physician-patient relationship.

### Dependence

The patient who is overly dependent on their healthcare team or system may defer decision-making to their clinician, frequently ask for reassurance, or refuse to carry out health-related tasks independently. Patients may exhibit these behaviors as a result of feeling overwhelmed or out of a desire to feel cared for. This can be difficult for the physician to avoid, as it appeals to their altruism and paternalism. However, it reinforces dependency and is fraught for the physician who is then responsible for the outcome, whether good or bad. It is valuable to recognize the patient’s emotional need underlying the dependence, which is connection and feeling cared for. Dependent patients are exquisitely sensitive to ruptures in the relationship.

The physician may initially be pleased with the patient’s adherence and positive regard, but over time may become exhausted by the reassurance-seeking behaviors. As the physician distances themself from the patient, dependent behaviors increase.

It can be helpful for the physician to approach the patient with a warm and supportive stance that neither challenges nor acquiesces to the dependency. Providing choices whenever possible can increase the patient’s sense of agency, gradually reducing dependent tendencies as the patient sees that asserting independence will not result in loss of the relationship. Inviting the patient to discuss their experience, as well as their hesitation to decide, may be useful. By setting boundaries while remaining warmly supportive, the physician can help the patient see that the emotional need can be met without the dependent behaviors.

In more extreme examples, the patient frequently returns to the hospital or seeks constant reassurance from physicians. In these cases, physicians may need to set firm boundaries and expectations; for example, limits on how long or how often they will see the patient.^44^ It may also be helpful to engage other support systems for the patient, including social work, chaplaincy, family, or friends.

## CONCLUSIONS

Difficult patient behaviors can impede care and worsen outcomes in acute medical settings. To improve care and reduce physician distress, it is helpful for physicians to be aware of these behaviors, to recognize their own countertransference, and to identify management strategies. Recognition that these behaviors serve a psychological purpose—typically to gain control over an uncertain or stressful situation or to alleviate distress—can reduce the physician’s frustration and provide insight on how to proceed. Attempts to engage the patient in other ways to meet these needs can be helpful. Additionally, setting limits and boundaries can be therapeutic, especially when these boundaries are enacted from a thoughtful and patient-centered perspective.

## References

[1] **Tyrer P, Reed GM, Crawford MJ.** Classification, assessment, prevalence, and effect of personality disorder. Lancet. 2015;385(9969):717-26. 10.1016/S0140-6736(14)61995-4 25706217

[2] **Huprich SK.** Personality pathology in primary care: ongoing needs for detection and intervention. J Clin Psychol Med Settings. 2018;25(1):43–54. 10.1007/s10880-017-9525-8 29322291

[3] **Hastrup LH, et al.** Societal costs of Borderline Personality Disorders: a matched-controlled nationwide study of patients and spouses. Acta Psychiatr Scand. 2019;140(5): 458-467. 10.1111/acps.13094 31483859

[4] **Sveen CA, et al.** Societal costs of personality disorders: A cross-sectional multicenter study of treatment-seeking patients in mental health services in Norway. J Clin Psychol. 2023. 10.1002/jclp.23504 36916214

[5] **Bailey J, Martin SA, Bangs A.** Managing difficult patient encounters*.* Am Fam Physician. 2023;108(5):494–500. 37983701

[6] **Jackson JL, Kroenke K. **Difficult patient encounters in the ambulatory clinic: clinical predictors and outcomes*.* Arch Intern Med. 1999;159(10):1069–75. 10.1001/archinte.159.10.1069 10335683

[7] **Hinchey SA, Jackson JL.** A cohort study assessing difficult patient encounters in a walk-in primary care clinic, predictors and outcomes. J Gen Intern Med. 2011;26(6):588-94. 10.1007/s11606-010-1620-6 PMC3101981 21264521

[8] **Jackson JL, Murphy MG, Fletcher KE.** The difficult inpatient, prevalence and characteristics*.* Patient Educ Couns. 2025;137:108785. 10.1016/j.pec.2025.108785 40311178

[9] **Enix C, et al.** Defusing disruption: A rapid qualitative analysis examining hospitalist experiences navigating behavioral escalation events. J Hosp Med. 2025; 20(9):963-970. 10.1002/jhm.70121 40611454

[10] **Steinmetz D, Tabenkin H.** The 'difficult patient' as perceived by family physicians*.* Fam Pract. 2001;18(5):495–500. 10.1093/fampra/18.5.495 11604370

[11] **Krieg A.** A contextual behavioral account of culture: example implementation of a functional behavioral approach to the study of cultural differences in social anxiety. Front Psychol. 2020;11. 10.3389/fpsyg.2020.00418 PMC7077519 32210900

[12] **Agarwal AK, et al.** Prevalence of behavioral flags in the electronic health record among black and white patients visiting the emergency department. JAMA Netw Open. 2023;6(1):e2251734. 10.1001/jamanetworkopen.2022.51734 PMC9857105 36656576

[13] **Burgess D, van Ryn M, Dovidio J, Saha S.** Reducing racial bias among health care providers: lessons from social-cognitive psychology. J Gen Intern Med. 2007;22(6):882-7. 10.1007/s11606-007-0160-1 PMC2219858 17503111

[14] **Caravella RA, et al.** Quality Improvement Framework to Examine Health Care Disparities in Behavioral Emergency Management in the Inpatient Medical Setting: a consultation-liaison psychiatry health equity project*.* J Acad Consult Liaison Psychiatry. 2023;64(4):322–31. 10.1016/j.jaclp.2023.04.002 37060945

[15] **Gabbard, GO.** The role of countertransference in contemporary psychiatric treatment. World Psychiatry. 2020;19(2):243–44. 10.1002/wps.20746 PMC7214951 32394567

[16] **Aasan OJ, Brataas HV, Nordtug B.** Experience of Managing Countertransference Through Self-Guided Imagery in Meditation Among Healthcare Professionals. Front Psychiatry. 2022;13:793784. 10.3389/fpsyt.2022.793784 PMC8891567 35250661

[17] **Book HE, Sadavoy J, Silver D.** Staff countertransference to borderline patients on an inpatient unit. Am J Psychother. 1978;32(4):521-32. 10.1176/appi.psychotherapy.1978.32.4.521 727308

[18] **Gabbard GO.** Splitting in hospital treatment. Am J Psychiatry. 1989;146(4):444-51. 10.1176/ajp.146.4.444 2648865

[19] **Wu T, et al.** Demystifying borderline personality disorder in primary care. Front Med (Lausanne). 2022;9:1024022. 10.3389/fmed.2022.1024022 PMC9668888 36405597

[20] **Blakeney EA, et al.** A scoping review of new implementations of interprofessional bedside rounding models to improve teamwork, care, and outcomes in hospitals. J Interprof Care. 2024;38(3):411-426. 10.1080/13561820.2021.1980379 PMC8994791 34632913

[21] **Aravind VK, Krishnaram VD, Thasneem Z.** Boundary crossings and violations in clinical settings. Indian J Psychol Med. 2012;34(1):21-4. 10.4103/0253-7176.96151 PMC3361837 22661802

[22] **Linklater D, MacDougall S.** Boundary issues. What do they mean for family physicians? Can Fam Physician. 1993;39:2569–73. PMC2379977 8292932

[23] **Haas LJ, Leiser JP, Magill MK, Sanyer ON.** Management of the difficult patient. Am Fam Physician. 2005;72(10):2063-8. 16342837

[24] **Loomis ME.** Nursing management of acting-out behavior. Perspect Psychiatr Care. 1970;8(4):168-73. 10.1111/j.1744-6163.1970.tb01320.x 5202520

[25] **Schuermeyer IN, et al.** Patients with challenging behaviors: Communication strategies. Cleve Clin J Med. 2017;84(7): 535-542. 10.3949/ccjm.84a.15130 28696194

[26] **Pam A.** Limit setting: theory, techniques, and risks. Am J Psychother. 1994;48(3):432-40. 10.1176/appi.psychotherapy.1994.48.3.432 7992873

[27] **Felde A.** ACLP How to Guide-Creating a Behavioral Plan. 2022; Available from: https://www.clpsychiatry.org/wp-content/uploads/ACLP-How-to-Guide-Creating-a-Behavioral-Plan.pdf.

[28] **Bass C, Wade DT.** Malingering and factitious disorder. Pract Neurol. 2019;19(2):96-105. 10.1136/practneurol-2018-001950 30425128

[29] **McDermott BE, Feldman MD.** Malingering in the medical setting. Psychiatr Clin North Am. 2007;30(4): 645-62. 10.1016/j.psc.2007.07.007 17938038

[30] **Margolis M, Wong TL, Shmuts R, Taylor JB.** Consultation-liaison case conference: a case of factitious disorder imposed on self*.* J Acad Consult Liaison Psychiatry. 2023;64(6):562–70. 10.1016/j.jaclp.2023.07.001 37499871

[31] **Marc D, Feldman GPY.** Dying to be Ill: True Stories of Medical Deception. 2018, New York, NY: Routledge.

[32] **Malone RD, Lange CL**. A clinical approach to the malingering patient. J Am Acad Psychoanal Dyn Psychiatry. 2007;35(1):13-21. 10.1521/jaap.2007.35.1.13 17480185

[33] **Bass C, Halligan P.** Factitious disorders and malingering: challenges for clinical assessment and management. Lancet. 2014;383(9926):1422-32. 10.1016/S0140-6736(13)62186-8 24612861

[34] **Taylor JB, Beach SR, Kontos N.** The therapeutic discharge: An approach to dealing with deceptive patients*.* Gen Hosp Psychiatry. 2017;46:74–8. 10.1016/j.genhosppsych.2017.03.010 28622821

[35] **Eldh AC, Ekman I, Ehnfors M.** Considering patient non-participation in health care*.* Health Expect. 2008;11(3):263–71. 10.1111/j.1369-7625.2008.00488.x PMC5060447 18816322

[36] **Eldh AC, Ekman I, Ehnfors M. **Conditions for patient participation and non-participation in health care*.* Nurs Ethics. 2006;13(5):503–14. 10.1191/0969733006nej898oa 16961114

[37] **Street RL Jr, et al.** Patient participation in medical consultations: why some patients are more involved than others. Med Care. 2005;43(10):960-9. 10.1097/01.mlr.0000178172.40344.70 16166865

[38] **Eldh AC, Ekman I, Ehnfors M. **A comparison of the concept of patient participation and patients' descriptions as related to healthcare definitions*.* Int J Nurs Terminol Classif. 2010;21(1):21–32. 10.1111/j.1744-618X.2009.01141.x 20132355

[39] **Carrese JA.** Refusal of care: patients' well-being and physicians' ethical obligations: "but doctor, I want to go home". Jama, 2006;296(6): 691-5. 10.1001/jama.296.6.691 16896112

[40] **Oh ES, Fong TG, Hshieh TT, InouyeSK.** Delirium in Older Persons: Advances in Diagnosis and Treatment. Jama, 2017;318(12):1161-1174. 10.1001/jama.2017.12067 PMC5717753 28973626

[41] **Vogel L.** Why do patients often lie to their doctors?. Cmaj. 2019;191(4):E115. 10.1503/cmaj.109-5705 PMC6342698 30692114

[42] **Palmieri JJ, Stern TA.** Lies in the doctor-patient relationship. Prim Care Companion J Clin Psychiatry. 2009;11(4):163-8. 10.4088/PCC.09r00780 PMC2736034 19750068

[43] **Mintz D.** Psychodyamic psychopharmacology: caring for the treatment-resistant patient. Washington, DC: American Psychiatric Association Publishing; 2022. pp. 61–62.

[44] **Levenson JL, G M, Muskin P.** Psychological responses to illness. In: The American Psychoatric Association Publishing Textbook of Psychosomatic Medicine. 2004.

[45] **Ward RK.** Assessment and management of personality disorders. Am Fam Physician. 2004;70(8):1505-12. 15526737

[46] **Angstman KB, Rasmussen NH.** Personality disorders: review and clinical application in daily practice. Am Fam Physician. 2011;84(11):1253-60. 22150659

[47] American Psychiatric Association. Personality Disorders, in Diagnostic and Statistical Manual of Mental Disorders. 2013: Washington, DC.

[48] **Martin J, et al.** The prevalence of personality disorder in a general medical hospital population in Jamaica. West Indian Med J. 2013:62(5):463-7. 10.7727/wimj.2013.066 24756662

[49] **Jeyasingam N, Jacob KS, Brodaty H.** Personality disorders in geriatric inpatients in a tertiary hospital. Australas Psychiatry. 2014;22(5):458–60. 10.1177/1039856214541688 25008096

